# Mesenchymal Stem Cell-Derived Exosomes in Cancer Resistance Against Therapeutics

**DOI:** 10.3390/cancers17050831

**Published:** 2025-02-27

**Authors:** Vignesh Balaji Easwaran, K Maya S Pai, K. Sreedhara Ranganath Pai

**Affiliations:** 1 Department of Pharmacology, Manipal College of Pharmaceutical Sciences, Manipal Academy of Higher Education, Manipal 576104, Karnataka, India; vigneshra96@gmail.com; 2 Department of Pharmacy Practice, Manipal College of Pharmaceutical Sciences, Manipal Academy of Higher Education, Manipal 576104, Karnataka, India; maya.mcopsmpl2023@learner.manipal.edu

**Keywords:** mesenchymal stem cells, exosomes, cancer, resistance, biomarkers

## Abstract

Mesenchymal stem cells (MSCs) are specialized cells that can differentiate into various types of cells. MSCs can be utilized to treat cancer. However, a MSC is considered a double-edged sword, because it can promote tumor progression and support cancer cell growth. Likewise, MSC-derived exosomes (MSC-Exos) carry various intracellular materials and transfer them to other cells. MSC-Exos could also cause tumor progression, including brain cancer, breast cancer, hepatic cancer, lung cancer, and colorectal cancer, and develop resistance against therapies, mainly chemotherapy, radiotherapy, and immunotherapy. An MSC-Exo promotes tumor development and causes drug resistance in various cancer types. The mechanisms involved in cancer drug resistance vary depending on the cancer cell heterogeneity and complexity. In this article, we have explained the various biomarkers and mechanisms involved in the tumor and resistance development through MSC-Exos in different cancer types.

## Simple Summary

Stem cell research has been growing widely in the biomedical science field. Various stem cells have been reported against diseases and have shown positive therapeutic efficacy. Mesenchymal stem cells (MSCs) are multipotent stem cells, meaning they can transform into different types of cells/tissues. Research on MSCs in cancer therapies is gaining more interest due to their potent action and fewer side effects. However, extracellular vesicles such as exosomes, tiny particles secreted from MSCs, can restrict the therapeutic function of MSCs against cancer disease and promote resistance. Molecular mechanisms involved in the tumor progression and development of resistance due to MSCs and MSC-derived exosomes have been explained in this article with pictorial representation. Understanding the functionality of MSCs and MSC-derived exosomes can help to prevent resistance and disease severity. Also, it may provide insight into the target/molecular pathways involved in the tumor progression due to MSCs or MSC-derived exosomes.

## 1. Introduction

Mesenchymal stem cells (MSCs) are adult stem cells capable of multilineage differentiation and self-renewal. MSCs are originally discovered in the bone marrow and later found in various tissues including muscle, peripheral blood, teeth, adipose tissue, the placenta, and the umbilical cord [1]. The origin and function of MSCs remain an active area of research worldwide. MSCs play a vital role in re-establishing and regulating homeostasis in the body [2]. Globally, cancer is a serious illness with a high mortality rate. After cardiovascular disease, cancer is regarded as the second leading cause of death in several countries. Because traditional therapies like radiation therapy and chemotherapy have drawbacks due to their toxicity profiles, the clinical management of cancer remains difficult. Unhealthy eating habits and a poor lifestyle are the main causes of cancer risk. Cancer is characterized by aberrant cell proliferation that can spread to other bodily areas. With around 10 million fatalities in 2020, it has emerged as a major cause of mortality worldwide. About 9.6 million individuals lost their lives to cancer in 2018 [3]. Lifestyle factors, including alcohol consumption and smoking, are considered crucial factors in the development of cancer and a primary target for cancer prevention [4]. Cancer is a hereditary illness that can result from both internal genetic alterations and the combined action of several external causes. Somatic DNA mutation and exposure to carcinogenic stimuli are the first steps in the cellular development of this malignant disease. Cancer can be classified based on the origin of the tissue or organ where the initial tumor forms [3].

Drug resistance in cancer remains a significant challenge in medical oncology. Certain processes that may hinder apoptosis, such as changes in drug transport and metabolism, the amplification and mutation of drug targets, and genetic rewiring, are adopted by malignant cells to withstand therapy. Both primary and secondary drug resistance could be caused by alterations in drug metabolism and the occurrence of resistance to one drug resulting in the development of resistance to another drug [5]. Cancer therapy faces various challenges in exerting the therapeutic effect and one of the obstacles is the development of resistance. The interplay between cancer cells associated with intrinsic and extrinsic factors causes resistance against cancer therapy. Intrinsic factors include genetic mutations, intracellular defense pathway activation, drug target alterations, desensitization, DNA repair enhancement, and tumor heterogeneity. The extrinsic factors relate to the tumor microenvironment, which is actively involved in the ability of cancer cells to escape from anticancer therapy [6]. MSC-derived exosomes (MSC-Exos) originate from plasma membranes in lipid raft microdomains, with sizes ranging from 40 nm to 160 nm. Exosomes can be released by their parent cells through invagination and budding. Exosomes could facilitate cell-to-cell communication through membrane fusion and endocytosis. Based on their biogenesis and functional properties, exosomes are divided into three different categories, including exosomes, apoptotic bodies, and microvesicles [7]. The morphological characteristics of MSCExos include a circular shape, with heterogeneous sizes observed under transmission electron microscopy. MSC-Exos could carry various intracellular particles such as lipids, proteins, nucleic acids (RNA, DNA, ncRNA), and other metabolites. These exosome cargos are involved in regulation of the physiological/therapeutic functions and induce pathological conditions. An MSC-Exo is crucially involved in drug resistance in targeted drug therapy, chemotherapy, immunotherapy, radiotherapy, etc. An MSC-Exo is directly involved in cancer therapy resistance because it transfers the functional RNAs and proteins [8]. Also, an MSC-Exo could cause tumor growth, invasion, and angiogenesis. An MSC-Exo influences the tumor microenvironment by transferring the metastasis signaling pathway and enhancing tumor progression. The physiological function of an MSC-Exo depends on the substance it mainly carries and releases into the systemic circulation. Various studies have reported that an MSC-Exo could promote tumor progression by transferring its intracellular substances and disturbing the functions. MSC-Exo-containing proteins and RNAs are associated with tumor drug resistance [9].

## 2. Molecular Mechanisms Involved in the Development of Resistance Against Treatments

### 2.1. Brain Cancer

Over the decades, surgical techniques, chemotherapy, and other adjuvant therapy have shown progressive development in the treatment of glioblastoma (GBM); however, the recurrence of the tumor mass is rapid due to several complex mechanisms, including the development of resistance against the treatment. It has been observed that MSH6 mutations are linked to resistance and recurrence in gliomas treated with chemotherapy such as temozolomide. These results most likely represent an escape from cell death as well as the accumulation of mutations driven by a malfunctioning MMR system [10]. Yu et al. reported that the overexpression of NFIA significantly promotes temozolomide (TMZ) resistance in GBM patients, and that the inhibition of NFIA through lentivirus reverses the development of resistance and tumorigenesis [11]. Also, a prolonged course or more than 3 courses of TMZ causes elevation of the O^6^-Methylguanine-DNA Methyltransferase (MGMT) gene, resulting in the development of TMZ resistance in GBM [12,13,14]. The autophagy process hijacked by tumor cells promotes drug and radioresistance and cancer progression. The dysregulated PTRF/Cavin-1 enhances the efflux of TMZ-mediated extracellular vesicles and causes drug resistance [15].

TMZ resistance is caused by three main DNA repair pathways, including the MGMT, MMR, and poly (ADP)-ribose polymerase (PARP) pathways. High MGMT expression is directly associated with the primary resistance mechanism against TMZ, while the MMR system in MGMT-deficient cells is associated with the secondary mechanism. The elimination of N7-methylguanine and N3-methyladenine adducts is the primary function of the poly(ADP-ribose) polymerase (PARP) pathway, which constitutes the third mechanism [16]. Smith et al. reported that the NANOG pathway contributes to chemoresistance in cancer stem cells (CSCs) through activation of the PI3K/AKT pathway in GBM [17]. Scherschinski et al. found that receptor tyrosine kinase AXL (RTK-AXL) plays a major role in therapy resistance against GBM. The endogenous administration of RTK-AXL promotes therapy resistance and it could be overcome by the combined treatment of radiation, TMZ, and tyrosine kinase inhibitors [18]. Lin et al. reported that the high expression of nucleobindin 2 (NUCB2) enhances radio resistance against GBM. Knockdown of NUCB2 in GBM 8401 and U87MG cells showed reduced colony formation after radiotherapy, whereas the overexpression of NUCB2 resulted in the development of resistance against radiotherapy in the GBM condition (Table 1) [19].

### 2.2. Lung Cancer

Lung cancer has a high incidence and a high fatality rate. KRas^G12C^ has revolutionized the treatment of patients with lung adenocarcinoma in clinical management. However, patients exposed to KRas^G12C^ inhibitors lead to the development of resistance. The ablation of KRas^G12C^ reverses the resistance and induces tumor regression. Also, the clinical efficacy of KRas^G12C^ inhibitors reduces due to the occurrence of resistance after a few months of treatment [20]. The innate and acquired resistance limits the cisplatin efficacy. The mechanism behind cisplatin resistance is complex. Galuzzi et al. categorized cisplatin resistance in four different ways, including the ineffective binding of cisplatin to DNA, on-target resistance, post-target resistance, and off-target resistance [21]. The acquired resistance is categorized into two major classes, epidermal growth factor receptor (EGFR)-dependent and EGFR-independent. Mesenchymal-epithelial transition (MET) amplification is the most common factor for acquired resistance to the first-line treatment osimertinib and it reduces the PFS of patients treated with EGFR-TKIs. Other cell cycle genes, including CCND1/2, cyclin-dependent kinase (CDK) 4/6, and CCNE1, have been associated with the intrinsic resistance to osimertinib [22]. EGFR alterations in the acquired resistance occur frequently at C797S. along with S768I, L718Q, and G796S. In most cases, EGFR-dependent resistance occurs at C797S and modifies the protein structure that hinders the covalent bond of osimertinib to mutant EGFR [22].

Most lung cancer patients develop two or more resistance mechanisms, either in temporal sequence or concurrently. Although spatially variable, MET amplification is seen in 66% (*n* = 6/9) of first-line osimertinib-treated patients and is linked to early progression. Osimertinib-resistance pathways that have been identified include programmed death-ligand (PD-L)-1, KRAS amplification, neuroendocrine differentiation without histologic change, and fusions of MKRN1-BRAF and ESR1-AKAP12 [23]. Acquired resistance occurs due to several mechanisms, as detected in 15 patients (14%), indicating that multiple mechanisms arise in 39% of all patients with an acquired resistance. The study revealed that MET amplification co-occurred with various major proteins including CDK6, BRAF (V600E), ALK fusion, EGFR 797S, HER2 amplification, and KRAS G12C. A common acquired resistance mechanism identified at the EGFR T790M mutation was detected in sixty-four (44%) patients, followed by the amplification of MET in nine (6%) patients and CDK6 in six (4%) patients [24]. Salgia et al. reported that the overexpression of ITGB4 promotes tolerance to sotorasib by enhancing the AKT-mTOR signaling pathway. Chronic treatment with sotorasib enhanced WNT expression and the β-catenin signaling pathway. Silencing both the β-catenin signaling pathway and WNT expression reduces the tolerance of sotorasib and increases sensitivity against the disease (Table 1) [25].

### 2.3. Hepatic Cancer

Hepatocellular carcinoma (HCC) is the leading cancer reported, with a high mortality rate. Studies have found that the elevated expression of FZD10 could promote lenvatinib resistance in liver cancer stem cells [26]. Studies reported that neurofibromin 1 (NF1) and dual specificity phosphatase 4 & 9 (DUSP4 & 9) are the crucial proteins causing resistance in HCC. The overexpression of fibroblast growth factor receptor (FGFR) 1 promotes lenvatinib resistance in HCC, caused by the downregulation of mTOR, ERK, and AKT signals. Lenvatinib resistance may also occur due to MET and miR-128-3p, which restrict the progression of the AKT apoptotic pathway and ERK cell cycle regulation [27]. In vivo studies, such as subcutaneous implantation, hydrodynamic injection, and orthotopic xenograft mouse models, have revealed that the upregulation of tRNA m^7^G methyltransferase-mediated WDR4 and METTL1 promote lenvatinib resistance in HCC [28]. Integrin beta-8 (ITGB8) is an integrin protein involved in lenvatinib resistance. Integrin promotes the molecular pathways involved in cell growth, such as the AKT-dependent signaling pathway. Also, ITGB8 is a significantly upregulated gene in HCC-resistant cells compared to Hep3B and Huh7 [29]. NF1 and DUSP9 are significantly related to lenvatinib resistance. NF1 triggers the PI3K-AKT and RAS MAPK pathways. These upregulated pathways degrade FOXO3, which leads to the development of tumors and resistance to lenvatinib. DUSP9 also causes resistance in lenvatinib treatment through the mitogen-activated protein kinase extracellular signal-regulated kinase (MAPK/ERK) pathway [30].

Wei and team showed that ZFP64 is a frequently upregulated protein in patients with anti-PD-1 resistance HCC. The overexpression of ZFP64 promotes anti-PD1 resistance by changing the macrophage polarization towards an alternative activation phenotype (M2) and advancing an inhibitory tumor microenvironment [31]. Activation of the Janus kinase/signal transducer, the activator of the transcription (JAK-STAT) pathway, oncogenic insulin-like growth factor (IGF/FGF), and KIF14 have been related to the development of sorafenib resistance. Also, the overexpression of epithelial-mesenchymal transition (EMT) and stemness contributed to the acquired sorafenib resistance in HCC organoids [32]. Xu reported that circRNA is upregulated in the sorafenib-resistant cells. Silencing circRNA by siRNA treatment could reduce the sorafenib resistance in HCC cells [33]. Under hypoxic conditions, the depletion of METTL3 promotes sorafenib resistance and induces angiogenesis in HCC cells, and triggers the autophagy-associated pathways. Moreover, m^6^A modification was reduced in the sorafenib-resistant HCC cells (Table 1) [34].

### 2.4. Colorectal Cancer

Colorectal cancer is second in terms of cancer-related mortality and the third most frequent kind of cancer globally. The EGFR-targeted therapy cetuximab has been approved for the treatment of RAS wild-type (WT) metastatic colorectal cancer. However, patients treated with cetuximab showed an innate resistance of around 60%. It is also reported that mutations in BRAF and PIK3CA develop resistance in cetuximab [35]. Likewise, Geogiou’s study revealed that hyperactivation of the PI3K pathway causes cetuximab resistance through KRAS/NRAS/BRAF^v600^ wild-type colorectal cancer [36]. Cetuximab, a monoclonal antibody-targeting EGFR remains unsuccessful, due to the development of rapid resistance in colorectal cancer (CRC). Russo et al. showed that macrophage migration inhibitory factor (MIF) triggers cancer resistance in CRC cells. Blocking MIF or the combined treatment of a MIF inhibitor and cetuximab could alleviate the resistance and increase the sensitivity to the CRC cells [37]. He et al. reported that 5-fluorouracil (5-FU) resistance was mediated through the CHK1 pathway. The aberrant expression of the Wnt signaling pathway disturbs CHK1 and causes 5-FU resistance in colorectal cancer. Overexpressing CHK1 in colorectal cancer cells increases the sensitivity of 5-FU and the knockdown of CHK1 showed the opposite effect in colorectal cancer [38]. Tage et al. showed that Visinin-like 1 in the Wnt/β-catenin signaling pathway is involved in the apoptosis resistance in CRC cells. The knockdown of Visinin-like 1 enhanced the apoptosis in CRC whereas the forced expression of Visinin-like 1 promoted the apoptosis resistance [39].

The resistance in metastatic CRC cells due to the mutation in BRAFV600E worsens the therapeutic effect of chemotherapy and reduces the overall survival rate. Dittmann et al. identified that overexpression of the Ezrin protein ultimately causes an acquired resistance against vemurafenib. The inhibition of Ezrin by NSC305787 promoted the pro-apoptotic effect and inhibited the proliferation of CRC cells [40]. Immune checkpoint inhibitors, a frontline treatment for many cancers, eventually leads to the development of resistance and causes tumor recurrence. Lv et al. found that reduced IFN-γ receptors cause anti-PD-1 resistance in the murine colorectal cancer model with decreased tumor-infiltrating lymphocytes [41]. Oxaliplatin resistance causes CRC recurrence and worsens the disease condition. Elevated circHIPK3 in CRC cells showed the development of chemoresistance in CRC patients. Mechanistically, sponging circHIPK3 with miR-637 promotes STAT3 expression and activates the downstream pathways, including the Bcl-2/Beclin1 signaling pathways [42]. The overexpression of miR-135b-5p in colorectal cancer may increase oxaliplatin resistance and trigger protective autophagy via the MUL1/ULK1 signaling pathway. A novel therapeutic approach for reversing oxaliplatin resistance in CRC may include targeting miR-135b-5p [43]. Clarke et al. showed that mutation in MEK1/2 or *phosphatase and tensin homolog* (PTEN) loss causes the acquired resistance against the MEK/PI3K inhibitor. In this condition, a low dose of the Bcl2 inhibitor navitoclax with the MEK-PI3K inhibitor showed a better synergistic effect and prevented resistance in CRC (Table 1) [44].

### 2.5. Breast Cancer

In the United States, breast cancer is the second most common cause of cancer-related death. In vitro and in vivo experiments have revealed that METTL3/YTHDF1 upregulates GPRC5A expression through m6A methylation. Further, GPRC5A promotes mTORC1/p70s6k by recruiting mTORC1 and consequently promotes resistance to docetaxel chemotherapy in triple-negative breast cancer (TNBC) cells, SUM159PT, and MDA-MB-231. It indicated that GPRC5A serves as an indicator/biomarker for TNBC and the associated liver metastasis. On the other hand, BCL2 overexpression enhances docetaxel resistance and suppresses the cytotoxic effect of the pro-apoptotic regulator BAX [45]. Tamoxifen is the first-line drug for premenopausal breast cancer patients, and it reduces the risk by ~50%. However, due to the development of resistance, tamoxifen does not show an effective reduction in tumor growth. It is reported that TMEM47 causes tamoxifen resistance in MCF-7 cells and the knockdown of TMEM47 reverses the resistance and promotes tumor cell death [46]. Likewise, Wang et al. reported that LncRNA H19 substantially upregulated in the tamoxifen-resistant breast cancer cells. The knockdown of LncRNA H19 could alleviate the autophagy in MCF-7 cells and prevent the resistance to tamoxifen [47]. Studies have reported that around 25% of HER2^+^ breast cancer patients developed resistance against trastuzumab. Ling et al. reported that circRNA circCDYL2 was overexpressed in trastuzumab-treated patients. Mechanistically, circCDYL2 increases the stability of GRB7 by preventing degradation through ubiquitination and allowing the interaction with FAK, which sustains the activities of ERK1/2 and AKT. Elevated levels of circCDYL2 cause low disease-free survival and overall survival. Also, they have reported that trastuzumab resistance could be reversed by GRB7 or FAK inhibitors in HER2^+^ breast cancer patients [48]. Also, immunotherapy targeting cancer-associated fibroblasts (CAF) could reduce the treatment resistance of HER2^+^ breast cancer [49].

Cyclin-dependent kinases 4 and 6 (CDK4/6) play a major role in the cell cycle and tumor development. CDK4/6 inhibitors showed tumor inhibition in breast cancer. However, the development of resistance against CDK4/6-like palbociclib remains challenging in clinical trials. Zhang et al. reported that high-throughput combinatorial drug screening and genomic sequencing revealed that microphthalmia-associated transcription factor (MITF) triggered O-GlcNAcylation by O-GlcNAc transferase (OGT) in palbociclib-resistant breast cancer cells. Mechanistically, the O-GlcNAcylation of MITF at Serine 49 promotes its interaction with importin α/β, thus activating its translocation to the nuclei, where it suppresses palbociclib-induced senescence. MITF inhibition or its O-GlcNAcylation re-sensitizes resistant cells to palbociclib [50]. Over the years, various resistance mechanisms have been reported, including the aberrant activation of insulin-like growth factor 1 receptor (IGF-1R), PI3K-AKT, MAPK pathways, metabolic dysregulation, upregulated PD-L1, mutation in EGFR, and the dysfunction of miRNAs. Also, PIK3CA may play a significant role in the development of resistance to trastuzumab and pertuzumab, which would worsen the metastasis progression in breast cancer [51]. Approximately one-third of estrogen receptor-alpha positive (ER^+^) breast cancers develop intrinsic or acquired resistance and tumor recurrence is promoted. It has been demonstrated that one of the main metabolic pathways in triple-negative BC (TNBC) that can trigger Src signaling is mitochondrial fatty acid β-oxidation (FAO). Ahn et al. reported that FAO inhibitors or knockdown ultimately reduced the resistance and enhanced the treatment efficacy in TNBC (Table 1) [52].

**Table 1:** Molecular biomarkers responsible for the development of resistance in various cancer types.

S. No.	Biomarker	Expression	Type of Resistance	Cancer Type	Reference
1	MSH6, PTRF/Cavin-1	Dysregulation	Temozolomide	GBM	[10,15]
2	NFIA, MGMT, PI3K/AKT	Overexpression	Temozolomide	GBM	[11,12,13,14,17]
3	NUCB2	Overexpression	Radioresistance	GBM	[19]
4	CCND1/2, CDK4/6, CCNE1, MET, EGFR, PD-L1, KRAS MKRN1-BRAF, ESR1-AKAP12	Amplification	Osimertinib	Lung Cancer	[22,24]
5	ITGB4, WNT/β-catenin signaling pathway	Overexpression	Sotorasib	Lung Cancer	[25]
6	FZD10, FGFR1, WDR4, METTL1, ITGB8	Overexpression	Lenvatinib	Hepatic Cancer	[26,29]
7	ZFP64	Overexpression	Anti-PD-1	Hepatic Cancer	[31]
8	JAK-STAT pathway, IGF/FGF, KIF14, EMT	Overexpression	Sorafenib	Hepatic Cancer	[32]
9	METTL3	Downregulation	Sorafenib	Hepatic Cancer	[34]
10	BRAF and PIK3CA	Dysregulation	Cetuximab	Colorectal Cancer	[35]
11	PI3K	Hyperactivation	Cetuximab	Colorectal Cancer	[36]
12	CHK1	Dysregulation	5-fluorouracil (5-FU)	Colorectal Cancer	[38]
13	Ezrin protein	Overexpression	Vemurafenib	Colorectal Cancer	[40]
14	IFN-γ receptor cause	Downregulation	Anti-PD-1 resistance	Colorectal Cancer	[41]
15	circHIPK3, miR-135b-5p	Overexpression	Oxaliplatin	Colorectal Cancer	[42,43]
16	MEK1/2 or PTEN	Dysregulation	MEK/PI3K inhibitor	Colorectal Cancer	[44]
17	mTORC1/p70s6k, BCL2	Overexpression	Docetaxel	TNBC	[45]
18	TMEM47, LncRNA H19	Overexpression	Tamoxifen	Breast Cancer	[46,47]
19	circRNA circCDYL2	Overexpression	Trastuzumab	HER2+ Breast Cancer	[48]
20	CDK4/6, O-GlcNAcylation by O-GlcNAc transferase (OGT)	Dysregulation	Palbociclib	Breast Cancer	[50]
21	IGF-1R, PI3K-AKT, MAPK, PD-L1, EGFR, miRNAs, PIK3CA	Dysregulation	Trastuzumab and Pertuzumab	Breast Cancer	[51]

## 3. MSC-Derived Exosomes in Cancer Resistance

MSC-derived exosomes play a crucial role in the development of resistance to cancer cells. MSC-derived exosomes cause drug resistance to promote tumor heterogeneity. Mediators in the cells such as RNA and proteins of MSC-derived exosomes cause drug resistance. Xu et al. reported that the bone marrow (BM)-MSC-Exo-associated activation of the PSMA3-AS1-PSMA3 pathway plays a role in the MM cells’ resistance to the proteasome inhibitor [53]. Likewise, miR-23b derived from BM-MSC-Exos was reported to cause resistance in the proteasome inhibitor, which is docetaxel in breast cancer [54]. Luo et al. showed that, mechanistically, MSC-Exos cause drug resistance in doxorubicin by inducing miR-21-5p and S100A6 against breast cancer [7,55]. Breast cancer cells develop their acquired resistance via MSC-Exo-secreted miRNA, miR-222/223 [8]. MSC-Exos from adipose impair the cisplatin in breast cancer by downregulating SLC9A1 via miR-1236. Upregulating SLC9A1 prevents the resistance mechanism by triggering the Wnt/β-Catenin signaling pathway [8]. MSC-derived exosomes cause chemoresistance signaling pathways including PI3K-AKT in pheochromocytoma in rat models [56].

**Figure 1:**
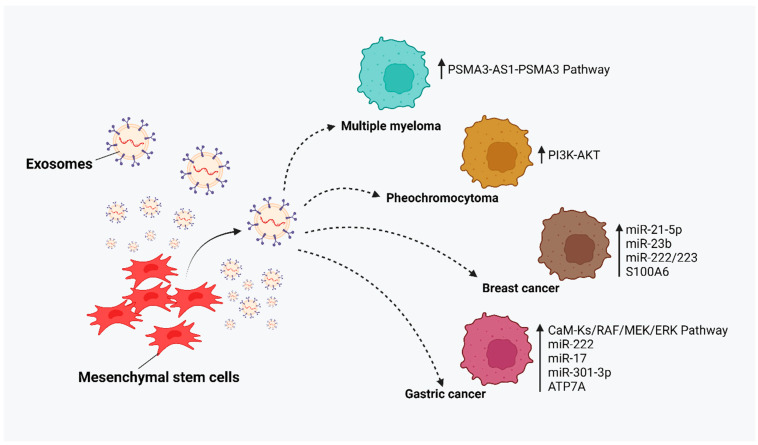
MSC-derived exosomes cause resistance in cancer cells.

MSC-Exos enhanced the expression of multidrug resistance proteins including MRP, MDR, and LRP by activating the RAF/MEK/ERK cascade and the calcium/calmodulin-dependent protein kinase which causes the resistance to fluorouracil in cancer treatment [57]. MSC-Exo-secreted CXCR4 and cell interactions in the tumor microenvironment promote resistance to tumor cells and prevent apoptosis [58,59]. MSC-derived exosomes induce drug resistance in 5-fluorouracil by activating the CaM-Ks/RAF/MEK/ERK pathway in gastric cancer. Also, it mediates through the drug efflux mechanism and promotes drug resistance to the recipient cells by transferring proteins and miRNAs such as ATP7A, miR-222, and miR-17 [60]. Zhu et al. observed that cisplatin/vincristine resistance occurs in gastric cancer cells due to the inhibition of the thioredoxin interacting protein (TXNIP) via the upregulation of miR-301-3p derived from MSC-Exos and the increased proliferation, migration, and suppression of the apoptosis in cancer [61]. MSC-derived exosomes could cause resistance in targeted therapy including tyrosine kinase inhibitors in acute myeloid leukemia, AML [62] (Figure 1).

## 4. MSC-Derived Exosomes in Tumor Progression

Bone marrow-originated mesenchymal stem cell-derived exosomes (BMMSC-Exos) contain a large number of oncogenic proteins, adhesion molecules, and cytokines and promote multiple myeloma. Also, BMMSC-Exos contain higher levels of CCL2 than the normal exosomes, which supports the MM growth [63]. Shi et al. reported that FGF19 was highly upregulated in MSC-Exos to activate the FGF19-FGFR4-dependent ERK signaling cascade and modulate EMT to promote nasopharyngeal carcinoma (NSC) [64]. Vallabhaneni et al. reported that MSC-Exos carry higher levels of tumor-promoting miRNAs such as miR-21 and miR-34a and proteins that trigger breast cancer progression [65,66]. Lin et al. reported that MSC-Exos induce a WNT signaling pathway and facilitate the migration and growth of breast cancer [67]. Qi et al. found that MSC-Exos trigger the Hedgehog signaling pathway in osteosarcoma and gastric cancer cells and cause tumor growth [68].

**Figure 2:**
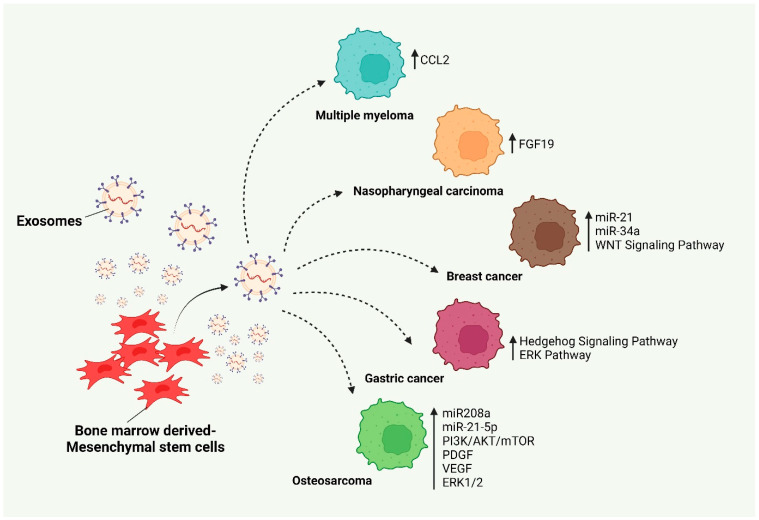
MSC-derived exosomes cause tumor progression.

Likewise, Huang et al. revealed that BMMSC-Exos enhance tumor progression by promoting oncogenic autophagy in osteosarcoma [69]. Qin et al. reported that BMMSC-Exos carry miR-208a and increase osteosarcoma viability, clonogenicity, and migration by activating ERK1/2 and downregulating programmed cell death (PDCD)-4 [70]. BMMSC-Exos carry a greater amount of miR-21-5p that significantly activates the PI3K/Akt/mTOR signaling pathway by reducing PIK3R1. Elevated levels of miR-21-5p cause increased proliferation and invasion in osteosarcoma cells [71]. The study reported that adipose MSC-Exos enhance angiogenesis due to the secretion of higher levels of platelet-derived growth factor (PDGF) and promotes tumor growth. Likewise, MSC-Exos stimulate angiogenesis by modulating vascular endothelial growth factor (VEGF), ERK1/2, and p38 in tumor cells [65,72]. Murine and human bone marrow-derived exosomes induce breast cancer cells towards bone marrow and survive as cancer stem cells (CSC) for a decade [73]. Also, MSC-Exos activate the ERK pathway and enhance the migration and proliferation of breast cancer in vitro [74]. MSC-derived exosomes promote tumor growth and cause drug resistance using various molecular mechanisms including the induction of AKT phosphorylation, EMT, vascular endothelial growth factor (VEGF), ERK1/2, and the WNT signaling pathway [75] (Figure 2).

## 5. Conclusions

An MSC-Exo is one of the potential agents that could promote drug resistance and restore tumor development. There are various molecular mechanisms involved in cancer drug resistance, especially with an MSC-Exo. An MSC-Exo transfers the intracellular particles from one cell to another which could enhance tumor progression and develop resistance. Molecular mechanisms such as PI3K, JAK-STAT, BRAF, etc are majorly involved in the drug resistance caused by MSC-Exos. Likewise, MSC-Exos also have unique cellular mechanisms to cause drug resistance and promote tumor growth. Understanding MSCs and MSC-Exos is complex because of their heterogeneous nature. Further research to explore the molecular mechanisms is required to prevent drug resistance and tumor growth.
